# Can Plasma Rich in Growth Factors Be Safe for Parental Use? A Safety Study in the Canine Model

**DOI:** 10.3390/ijms19092701

**Published:** 2018-09-11

**Authors:** Elena Damiá, Deborah Chicharro, Mónica Rubio, José María Carrillo, Joaquín Sopena, Belén Cuervo, Sergio López, José Manuel Vilar

**Affiliations:** 1 Bioregenerative Medicine and Applied Surgery Research Group, Department of Animal Medicine and Surgery, Faculty of Veterinary, Universidad Cardenal Herrera-CEU, CEU Universities, 46115 Valencia, Spain; elena.damia@uchceu.es (E.D.); debora.chicharro@uchceu.es (D.C.); mrubio@uchceu.es (M.R.); jcarrill@uchceu.es (J.M.C.); jsopena@uchceu.es (J.S.); belen.cuervo@uchceu.es (B.C.); 2 García Cugat Foundation CEU-UCH Chair of Medicine and Regenerative Surgery, 08006 Barcelona, Spain; 3 Department of Animal Pathology, Instituto Universitario de Investigaciones Biomédicas y Universitarias, Universidad de Las Palmas de Gran Canaria, 35416 Trasmontaña S/N. Arucas, Spain; sergiolopezbarbeta@gmail.com

**Keywords:** platelet-rich plasma (PRP), plasma rich in growth factors (PRGF), insulin-like growth factor-1 (IGF-1), canine

## Abstract

Low invasiveness is the main goal of modern surgery. The use of platelet-rich plasma (PRP) is known to be effective in a variety of applications, such as oral, maxillofacial, orthopedic, dermatologic and cosmetic surgeries. However, a potential ergogenic and carcinogenic effect of PRP derivatives by means of the insulin-like growth factor-1 (IGF-1) pathway has been suggested. Because of this notion, the purpose of this study is to assess the effect of a commercially available PRP-derivative intramuscular injection in the lumbar muscular tissue (local effect) and to determine the IGF-1 blood concentration (systemic effect) on healthy beagle dogs. Local effect was evaluated by computed tomography (CT) scan and echography, and systemic effect was calculated by blood testing on days 0, 14, 28, 42 and 56. No statistically significant changes were observed; thus, PRGF could be considered safe when using therapeutic doses.

## 1. Introduction

The main goal of modern surgery is to reduce invasiveness and increase the healing process. Regenerative medicine is now one of the most attractive and interesting disciplines that aims to regenerate or repair damaged tissues [1]. Platelet-rich plasma (PRP) is currently used in different medical fields and involves a minimum risk of immune reactions and transmission of diseases [2]. The first descriptions of the development and use of PRP were in the early 1970s in the hematology field, followed by maxillofacial and oral surgery [3]. Subsequently, PRP has been used in a wide variety of disciplines, such as aesthetic dermatology [4], including alopecia [5] and skin rejuvenation [6]; the musculoskeletal field [7]; oral and maxillofacial surgery [8]; and ophthalmology [9].

In recent years, the use of regenerative therapies, such as plasma rich in growth factors (PRGF), a PRP derivative, is also gaining interest to promote healing in muscle injuries, and consequently, to enable the patient to resume daily and sports-related activities quickly without relapse. A considerable number of authors have reported that growth factors (GFs) and fibrin matrix are crucial for the muscle repair and regeneration process by promoting myogenesis, angiogenesis and fibrogenesis [10,11], and promising results have been proven with this novel biological approach in managing musculoskeletal pathologies [12].

Besides the possible beneficial effects of PRP derivatives, several concerns have been raised regarding undesirable side effects. Some authors describe a potential carcinogenetic effect related to the insulin-like growth factor-1 in humans (IGF-1) [13,14,15,16]. In veterinary medicine, some studies demonstrated IGF-1 receptor expression and its role in canine osteosarcoma [17] and mammary gland carcinoma [18]. In this sense, there are different IGF-1 isoforms, such as IGF-1Ea, IGF-1Eb and IGF-1Ec. The IGF-1Ec isoform has an important role in physiology and cancer biology through its Ec peptide. After the tissue is damaged from mechanical stimuli, IGF-1Ec isoform and the Ec peptide levels are induced in the muscle, tendon and bone, and its secretion produces cellular proliferation [18,19,20].

Multiple GFs are secreted during muscle repair and hypertrophy, but only IGF-1 and its isoforms participate in muscle proliferation, differentiation and regeneration [21]. It has been demonstrated that IGF-1, at the onset of the mechanical stress on human skeletal muscle cells, increases IGF-1Ec isoform [22]. Moreover, the application of the Ec peptide in a rat model provided an increase in the expression of myofibroblasts in wound healing [23]. Overall, published scientific research supports that GFs included in PRP are unlikely to trigger a potent ergogenic effect. Regarding IGF-1, the doses in PRP are subtherapeutic, only 1% of the total IGF-1 is biologically available. IGF-1 in plasma has a low half-life (20 h) in humans [24], and although we could not find published data, dogs should be similar. To demonstrate this controversy, the use of PRP intramuscular injections in athletes was prohibited by the World Anti-Doping Agency (WADA) in 2010, despite the use of these biological therapies as the “gold standard” for muscle injury treatment, but again permitted in 2011 due to the limited evidence for systemic ergogenic effect of PRP. Nevertheless, the use of individual GFs in athletes continues to be prohibited under Section S2 of the 2018 WADA Prohibited List [25], particularly fibroblast growth factor (FGF), hepatocyte growth factor (HGF), insulin-like growth factor (IGF-1), platelet-derived growth factor (PDGF) and vascular endothelial growth factor (VEGF), because of concerns regarding their abuse as ergogenic substances [26].

For these reasons, along with the multiple applications of these therapies and several pieces of evidence for specific ergogenic (local) and carcinogenic (systemic) effects, the use of PRGF in muscle tissue remains of great interest.

Based on this, the purpose of our study was to evaluate (a) the local effect, measuring the cross-sectional area (CSA) of the lumbar muscles by using imaging systems, and (b) the systemic effect by blood IGF-1 determination in healthy beagle dogs, which were submitted to a PRGF intramuscular lumbar injection.

## 2. Results

### 2.1. Animals

The ages of the animals included in the study were (mean ± SD) 73.5 ± 38.8 months in the control group, 76.5 ± 38.7 months in the PRGF group, and 79.4 ± 38.9 months in the triple dose (HPRGF) group. Their weights were 16.9 ± 3.3 kg in the control group, 14.8 ± 3.1 kg in the PRGF-treated dogs, and 14.9 ± 2.5 kg in the HPRGF group.

### 2.2. IGF-1 Evaluation

No statistically significant differences were observed along the studied times nor between groups in IGF-1 serum concentrations. As a result, IGF-1 serum concentrations remained stable throughout the study, showing the inability of PRGF intramuscular injection to have a systemic effect (Table 1).

Regarding external factors affecting IGF-1 serum concentrations, in the three studied groups, weight influenced IGF-1 serum concentrations. In this way, the control group showed higher serum concentrations due to a larger weight in the animals during this phase. Conversely, older animals had lower IGF-1 serum concentrations compared to younger animals.

### 2.3. Computed Tomography and Echography Evaluation

Both CT-scan and echography images were carried out between the three studied groups and along the studied times. A correlation test was also realized between the two measures. No statistically significant differences were obtained between groups nor along the studied times in the studied anatomic level (L5) (Table 2, Table 3 and Table 4). As a result, no local effect and, therefore, no muscular hypertrophy were observed after PRGF injection.

## 3. Discussion

The aim of the present study was to determine if an intramuscular injection of PRGF increases circulating levels of the potentially ergogenic growth factor IGF-1 and thus induces skeletal muscle hypertrophy and, in the last instance, cancer.

Assuming that injections of PRGF within the injured muscle enhance healing and functional recovery [27], the question remains as to what is the correct dosage. In humans, Hamilton et al. (2010) demonstrated that a single injection of 3 mL PRP was effective for grade II semimembranosus strain injury, with a full recovery after 17 days post-injection [28]. Moreover, Hamid et al. (2012) used the same dose after a grade II hamstring injury, and the time needed to return to play in participants was 16 weeks [29]. In this sense, we decided to use a single injection of 1 mL of PRGF (normal-dose PRGF; PRGF group) and 3 mL of PRGF (high-dose PRGF; HPRGF group), taking into account the differences in size and weight between humans and dogs.

Particular attention is drawn to IGF-1 due to its potential ergogenic [14] and carcinogenetic effects [27]. In our study, a single intramuscular PRGF injection in healthy beagle dogs has no effect on circulating IGF-1 values, even when the standard PRGF concentration was increased three-fold. In reference to this systemic anabolic action of PRGF and in concordance with our results, several studies have also demonstrated that different commercial PRP systems do not increase IGF-1 concentrations over normal circulated blood levels [28,29,30]. Moreover, further scientific research supports the opinion that PRP is unlikely to promote an ergogenic effect in patients. This is due to subtherapeutic doses of IGF-1 in PRP. The isoform of IGF-1Ec in PRP is the isoform that causes muscular hypertrophy [31]. The unbound IGF-1 has too-short a half-life to exert systemic effects, and only 1% of IGF-1 is biologically available and active [32].

The last undesirable effect of IGF-1 suggested by other authors is a potential carcinogenic effect in humans [16,33] and in veterinary medicine [17,18]. Some authors [31,34] have suggested that growth factors, acting only on cell surface receptors, do not access the cell and do not promote cell DNA mutation. In agreement with Schippinger et al. [19], in our study neither the PRGF or HPRGF intramuscular injection showed an increase in IGF-1 serum concentrations. This suggests that PRGF application can be considered a safe method of treatment after 14 days, 28 days, 42 days and 56 days post-injection. Although long-term effects of multiple injections of PRGF were not examined in our study, the HPRGF used in one of the groups contained three-times the dose of normal PRGF, and no statistically significant differences were shown, suggesting that several applications over time would not alter IGF-1 circulating levels [35].

In reference to the effect of weight on serum IGF-1 concentrations, a positive correlation was observed [36], where animals from the control group had higher IGF-1 levels due to a greater weight. In the same way, high IGF-1 concentrations have been shown in obese dogs, which return to normal levels after weight loss [37]. Moreover, regarding the influence of age on IGF-1 circulating levels, with the exception of the control group, older animals show lower systemic IGF-1 concentrations. Moreover, our results are in agreement with other studies in humans [38] and veterinary medicine [39].

To assess the evolution of muscular fiber size after a PRGF injection, an imaging study was carried out with infiltrated lumbar muscles by echography and CT scan. No statistically significant differences were found between groups regarding muscle area measurements. As a result, the muscle size was similar in both infiltrated areas after intramuscular PRGF, HPRGF or saline solution, showing that intramuscular PRGF does not exert an anabolic effect even when injecting high doses.

## 4. Materials and Methods

### 4.1. Animal Model

A total of 24 healthy adult Beagle dogs were used in this study and were divided into three groups of eight dogs, five males and three females in each group, with ages ranging from 3–4 years and weights from 10–18 kg. Complete physical examination, haematology, and serum biochemical analyses were performed to ensure that animals were healthy.

The study protocol was approved by the Ethics Committee for Animal Welfare at the University CEU-Cardenal Herrera of Valencia (CEBA/2013).

### 4.2. Plasma Rich in Growth Factors (PRGF) Preparation and Infiltration

PRGF^®^-Endoret^®^ technology (BTI Biotechnology Institute, Álava, Spain) was followed to obtain an autologous preparation of PRP [29]. Briefly, blood was collected from the external jugular vein of each dog under sterile conditions in Vacutainer sodium citrate 3.8% tubes (Blood-Collecting Tubes^®^, BTI Biotechnology Institute, Álava, Spain). The tubes were centrifuged at 460× *g* for eight minutes (PRGF^®^ System III, Biotechnology Institute, Álava, Spain) to separate the different blood phases. The fraction located immediately above the buffy coat (white fraction) corresponded to PRGF, which was activated by adding 5% of calcium chloride (CaCl_2_ 10%) just before infiltration to activate platelets for GF release.

After obtaining PRGF, the platelet concentrations and the presence of leukocytes between whole blood, PRGF, and plasma poor in growth factors (PPGF) were compared on the initial day of each of the 3 study groups. Regarding the concentration of platelets, in the 3 study groups, the authors observed an increase in the number of platelets between the blood, the PPGF, and the PRGF, showing PRGF platelet values of 1.5–2-times higher than the concentration in blood and PPGF, according to what has been previously described [29]. With regard to the concentration of leukocytes, there are statistically significant differences between blood, PRGF, and PPGF in the three groups of the study. These results confirm the absence of white blood cells after the centrifugation of the blood and the separation of the different types of plasma. These results coincide with a previous report [30], which defends the absence of leukocytes in the PRGF.

Every dog was injected in the left lumbar muscles (lumbar multifidus, latissimus dorsi lumbar, and iliocostal lumbar muscles) at the 5th lumbar vertebrae level with the following treatments:

- Treatment 1: single dose of 1 mL sterile saline solution activated with 0.05 mL CaCl_2_ 10% (control group) [40].
- Treatment 2: single dose of 1 mL PRGF activated with 0.05 mL CaCl_2_ 10% (PRGF group).
- Treatment 3: single dose of 3 mL PRGF activated with 0.15 mL CaCl_2_ 10% (HPRGF group).

### 4.3. Determination of IGF-1 Concentrations

Under sterile conditions, blood samples were collected from the external jugular vein after intramuscular sedation with medetomidine (0.01 mg/kg), morphine (0.2 mg/kg), and midazolam (0.2 mg/kg). Samples were obtained at baseline, and 14 days, 28 days, 42 days and 56 days after injection of intramuscular PRGF. IGF-1 was analyzed by automated immunoassay system (Immulite 1000 IGF-1 assay; Diagnostic Products, Los Angeles, CA, USA) previously validated in dogs [41].

To evaluate the local effect of the intramuscular PRGF injections, ultrasound and CT-scan studies were performed.

### 4.4. Muscle Tissue Evaluation by Echography

Following the previous suggestions by other authors that echography has equal sensitivity to MRI for acute muscle injury (hamstring muscle) especially when performed within 2 weeks following injury [42], the ultrasound study was performed for each group (Control, PRGF, HPRGF) at baseline, and 14 days, 28 days, 42 days and 56 days after injection of the corresponding treatments.

**Figure 1:**
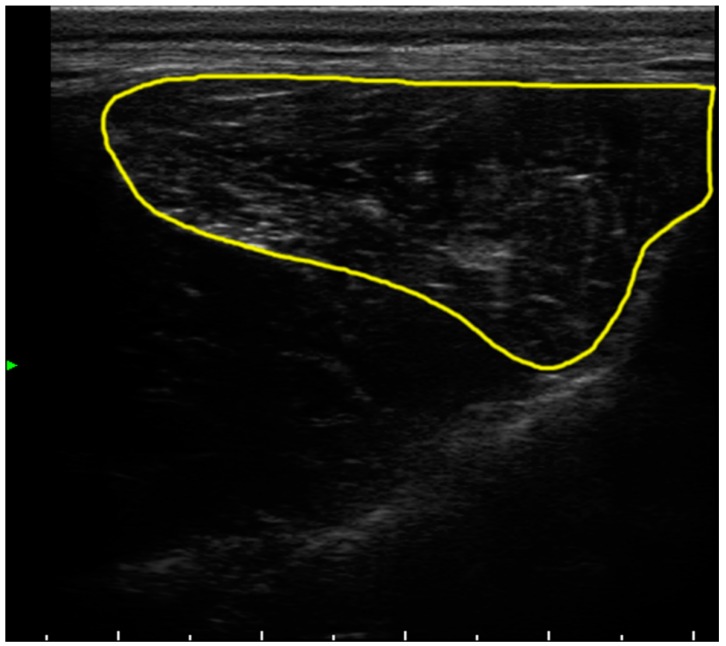
Ultrasonogram of one dog. The approximate contour of the measured lumbar area is delineated in yellow.

The ultrasound images (Esaote mylab60, Genoa, Italy) were taken at left L5 level (midpoint 5th lumbar vertebrae) to calculate the muscular area average (Figure 1).

### 4.5. Muscle Tissue Evaluation by Computed Tomography

A CT scan (CT-max, General Electric, Madrid, Spain) was performed every 14 days within the study; therefore, measurements were taken at baseline, 14, 28, 42, and 56 days (i.e., the same as ultrasound examination) under sedation with medetomidine (0.01 mg/kg), morphine (0.2 mg/kg), and midazolam (0.2 mg/kg).

**Figure 2:**
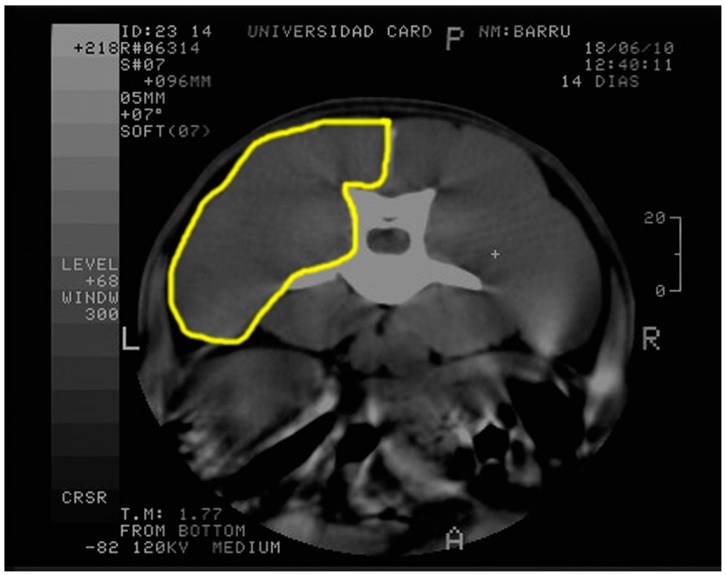
CT scan of one dog. The approximate contour of the measured lumbar area is delineated in yellow.

CT-scan images were performed at the same anatomic level as the ultrasound study, and three corresponding measurements were taken from lumbar muscles at left side (Figure 2).

### 4.6. Image Processing

Once the ultrasound and CT-scan images were collected, the contours were traced. Measurements from the designated muscular lumbar area were determined via quantitative morphometry using Image Pro Plus software (for Windows 2000, Silver Spring, MD, USA). The median value of the three measurements was considered as long as the measurements differed <10%. When the difference was >10%, new measurements were obtained.

### 4.7. Statistical Analysis

The data were processed using the SPSS 15.0 for Windows (Chicago, IL, USA). A descriptive study of the mean, standard deviation, and confidence intervals was made for each variable. A value of *p* ≤ 0.05 was considered significant. The result of each parameter was evaluated with a nonparametric Kolgomorov–Smirnov test for normality and log transformed if necessary. ANOVA repeated-measures and post-hoc Tukey tests were performed to assess differences with the baseline. A one-way ANOVA was conducted each time, to assess differences between groups, and a post-hoc Tukey test was carried out when necessary. A Pearson correlation between echography and CT-scan measures was obtained.

## 5. Conclusions

A single intramuscular application of PRGF does not significantly increase systemic IGF-1 levels nor increase muscle mass, even when three-times the normal dose in canine species was used. Therefore, in the canine species, a single application of PRGF is safe for parental use with respect to local and systemic IGF-1 levels and cancer risk. Despite this, further studies are needed to prove and evaluate the safety of this therapy in humans.

## Figures and Tables

**Table 1:** IGF-1 measurements (ng/mL) in the three study groups.

Time	Group	Mean	SD
**Baseline**	Control	166.3	31.1
	PRGF	132.7	49.1
	HPRGF	95.6	31.5
**14 days**	Control	174.7	25.1
	PRGF	114.5	40.2
	HPRGF	117.1	30.4
**28 days**	Control	157.9	26.4
	PRGF	133.8	46.7
	HPRGF	127.5	34.0
**42 days**	Control	134.6	20.0
	PRGF	143.9	48.5
	HPRGF	126.2	33.1
**56 days**	Control	155.6	24.3
	PRGF	139.4	49.9
	HPRGF	117.2	38.5

IGF-1: Insuline Growth Factor-1; PRGF: single dose of Plasma Rich in Growth Factors; HPRGF: triple dose of Plasma Rich in Growth Factors.

**Table 2:** Ultrasound measurements of the muscular area at left L5 level in the three study groups.

Time	Group	Mean	SD
**Baseline**	Control	12.8	3.6
	PRGF	12.5	3.1
	HPRGF	12.8	3.5
**14 days**	Control	13.4	3.3
	PRGF	12.6	3.0
	HPRGF	13.1	3.3
**28 days**	Control	13.1	3.2
	PRGF	12.7	3.0
	HPRGF	13.3	3.1
**42 days**	Control	13.0	3.1
	PRGF	12.6	3.1
	HPRGF	13.0	3.1
**56 days**	Control	13.0	3.1
	PRGF	12.4	3.1
	HPRGF	12.8	3.2

L5: fifth lumbar vertebra.

**Table 3:** CT-scan measurements of the muscular area at left L5 level in the three study groups.

Time	Group	Mean	SD
**Baseline**	Control	13.3	2.6
	PRGF	12.7	2.4
	HPRGF	12.8	2.9
**14 days**	Control	12.9	2.7
	PRGF	12.2	2.5
	HPRGF	13.2	2.8
**28 days**	Control	12.5	2.5
	PRGF	12.3	3.3
	HPRGF	12.8	2.6
**42 days**	Control	12.4	2.5
	PRGF	12.1	2.7
	HPRGF	13.1	3.1
**56 days**	Control	12.9	2.6
	PRGF	11.8	2.4
	HPRGF	12.4	3.1

**Table 4:** Pearson correlation between ultrasound and CT-scan measurements.

Correlations			
		L5 US	L5 CT scan
L5 US	Pearson correlation	1	0.928 *
	Sig. (2-tailed)		0.000
	*N*	210	105
L5 CT scan	Pearson correlation	0.928 *	1
	Sig. (2-tailed)	0.000	
	*N*	105	105

* Correlation is significant at the 0.01 level (2-tailed).

## Abbreviations

PRGF	plasma rich in growth factors
HPRGF	high-dose plasma rich in growth factors
PRP	platelet-rich plasma
IGF-1	insulin-like growth factor-1
CRP	C-reactive protein
WADA	World Anti-Doping Agency
VEGF	vascular endothelial growth factor
FGF	fibroblast growth factor
HGF	hepatocyte growth factor
PDGF	platelet-derived growth factor
